# Suicidal Ideation, Attempt, and Associated Factors among Patients with Tuberculosis in Ethiopia: A Cross-Sectional Study

**DOI:** 10.1155/2019/4149806

**Published:** 2019-03-13

**Authors:** Alemayehu Molla, Atikilt Mengesha, Habtamu Derajew, Habtamu Kerebih

**Affiliations:** ^1^Department of Psychiatry, College of Health Science, Dilla University, Dilla, Ethiopia; ^2^Amanuel Mental Specialized Hospital, Addis Ababa, Ethiopia; ^3^Departments of Psychiatry, School of Medicine, College of Medicine and Medical Sciences, University of Gondar, Gondar, Ethiopia

## Abstract

**Background:**

Suicidal behaviors among people with tuberculosis are one of the commonest psychiatric emergencies that need a major public health concern. People with tuberculosis show suicidal ideation and attempt, which are problems to end life. In Ethiopia large numbers of people are affected by tuberculosis. Therefore, assessing suicide among patients with tuberculosis is important in implementing further interventions.

**Methods:**

An institutional based cross-sectional study was conducted among systematic random samples of 415 and face-to-face interview was used. Suicidal ideation and attempt were assessed by using suicidality module World Health Organization (WHO) composite International diagnostic interview (CIDI). Data was analyzed by using SPSS version 20. Bivariate and multivariate binary logistic analyses were done to identify associated factors to both suicidal ideation and attempt. P values less than 0.05 were considered statistically significant and strength of the association was presented by adjusted odds ratio with 95% C.I.

**Results:**

The prevalence of suicidal ideation and attempt among tuberculosis patients was 17.3% (95%CI, 13.7-20.6) and 7.5 %( 95%CI, 4.8-10.4), respectively. Being female (AOR=2.7, 95% CI 1.39, 5.23), no formal education (AOR=3.35, 95%CI 1.26, 9.91), extra-pulmonary tuberculosis (AOR=2.35, 95%CI 1.1, 4.98), depression (AOR=4.9, 95%CI, 2.56, 9.4), and perceived TB stigma (AOR=3.24, 95%CI, 1.64, 6.45) were statistically associated with suicidal ideation. Factors like being female (AOR=4.57, 95%CI, 1.7, 12.27), MDR-TB (AOR=3.06, 95%CI, 1.23, 7.57), comorbid HIV illness (AOR=6.67, 95%CI, 2.24, 19.94), and depression (AOR=4.75, 95%CI, 1.89, 11.91) were associated with suicidal attempt.

**Conclusion:**

Developing guidelines and training of health workers in TB clinics is important to screen and treat suicide among patients with tuberculosis.

## 1. Background

Suicide is a fatal act of terminating one's own life. It is a complex process that involves a series of pathways and mechanisms from initiation of ideation to finally attempting suicide [1]. Some plan for days, weeks, or even years before acting while others take their lives impulsively without premeditation [2]. Suicidal ideation is an important phase in the suicidal process preceding attempted suicide which is the major risk factors for completed suicide [2, 3]. Suicide is a significant public health problem. It is the 10th leading cause of death worldwide and the second leading cause of death among people aged between 15 and 29 years [4]. The World Health Organization (WHO) 2012 report indicated that more than 804 000 suicide deaths occurred worldwide per year and suicide represents 1.8% of the global burden of disease [5]. The global burden of suicide is estimated to increase to 2.4% by the year 2020 and the rate of death due to suicide will be one for every 20 seconds [3].

Among others chronic illnesses such as tuberculosis (TB) increases the risk of suicide. TB is chronic infectious disease caused by the* Mycobacterium tuberculosis* which is one of the leading causes of morbidity and mortality worldwide [6]. Despite its global nature, approximately 85% of TB cases reside in Asian and African countries [7]. According to WHO 2013 global TB control report, Ethiopia ranks 7th of the 22 highest TB burden countries in the world. The country is second in Africa and 15th among 27 countries with high burden multi drug resistant (MDR) TB with an estimated number of 5200 cases occurring each year [8, 9].

Various study survey results have shown that suicidal ideations and attempts among people with chronic illnesses are very common and higher than general population. A study from United States reported that in patients with at least one general medical illness suicidal ideation was 25.2% while suicide attempt was 8.9% [10], whereas a survey from Korea among Chronic Obstructive Lung Disease (GOLD) patients indicated that suicidal thoughts were reported by 16.0% to 23.8% of patients at different severity level of the disease [11]. Studies focused on TB alone also indicated an increased suicidal ideation and attempt. In a South African study, 9% of TB patients were reported to have suicidal ideation and 3.1% of them attempted suicide [12]. A large number of people living with MDR TB were reported to have suicidal ideation (44.3%) and attempt suicide [14.8%] in a study result from Nigeria [13].

Female sex, stigma related to TB, HIV coinfection, depression, extra pulmonary TB, and treatment category of MDR TB were the factors identified as being associated for suicide among patients with TB in previous study results [10–15].

Even though suicidality is common in developing and developed countries with chronic medical problems, little attention is paid to it outside psychiatric settings [3, 4, 16]. In Ethiopia suicide is studied with severe mental illness and other chronic conditions and showed high magnitude [17, 18]. But attention was not paid to identifying suicide among patients with TB which is common chronic health problem in the country. Therefore, this study was conducted to assess magnitude of suicidal ideation, attempt, and associated factors among patients with tuberculosis.

## 2. Methods

### 2.1. Study Design and Setting

Institutional based cross-sectional study was conducted from May 7 to June 29, 2018, at St Petros TB Specialized Referral Hospital. The hospital is one of the first TB hospitals and a referral hospital in Ethiopia dedicated to treating both drug susceptible and resistant types of TB. The hospital provides both inpatient and outpatient TB treatment service. Even though it is specialized by TB, hospital is giving other essential services for large number of population. For the outpatient service an average of 7 MDR and 16 non-MDR tuberculosis patients visit hospital per day.

### 2.2. Study Participants

All adult patients with tuberculosis attending outpatient TB treatment were the study participants. Patients with age of 15 years and younger, patients receiving inpatient treatment and critically ill patients with difficulty of communication were excluded.

### 2.3. Sampling

About 423 samples were recruited for the study using single population proportion formula considering proportion of suicide as 50% among TB patients, 5% margin of error, 95% confidence level, and 10% nonresponse rate. Systematic random sampling technique was used to select participants and finally 415 (308 non-MDR-TB and 107 MDR-TB) patients interviewed.

### 2.4. Instruments

Data was collected by two trained psychiatric nurses by using pretested face to face interviewer administered questionnaires. Depression was assessed using PHQ-9 questionnaires which has 9 items and each item has 4-point Likert scale (0=not at all to 3=nearly every day). Score range is 0-27 and individuals who score 10 and above were considered as having depression [19]. The Cronbach alpha of PHQ-9 in the current study was 0.87. Social support was collected using Oslo-3 item social support scale. The sum score scale ranging from 3 to 14 which has 3 categories: poor support 3-8, moderate support 9-11, and strong support 12-14 [20]. Cronbach alpha of Oslo-3 in current study was 0.85. Stigma related to TB was collected using 12-item perceived TB stigma scale. Perceived TB stigma scale is 12-item scale which is used to assess stigma felt by TB patients. This stigma scale consists of four-point Likert scale from strongly disagree, disagree, agree, and strongly agree. Item scores of the stigma questions were summed to construct a single stigma variable and participants were classified as having or not having perceived stigma using the mean of the stigma as a cut-off point [21]. The internal consistence, Cronbach alpha, of the 12-item perceived TB stigma scale in current study was 0.92.

The outcome variables of the current study, suicidal ideation and attempt, were assessed by using suicidality module of World Mental Health (WMH) survey initiative version of the World Health Organization (WHO) composite International diagnostic interview (CIDI) in which suicide is studied among patients with HIV/AIDS [18, 22], epilepsy [23], and severe mental illness [17] in Ethiopia. The tool's local language Amharic version is validated in Ethiopia at both clinical and community settings [24, 25]. Its internal consistence, Cronbach alpha, in current study was 0.87.

### 2.5. Data Quality Control

To produce quality data, the study utilized validated assessment tools and in a local language. Data collectors were trained on assessment tools and how to collect data using the tools. Pretest was also done before the actual study on 5% of the people who had similar characteristics with the study participants. Data was also double entered in SPSS and data checked and corrected before the statistical analysis.

### 2.6. Statistical Analysis

Data was analyzed by using statistical package for social science (SPSS) window software version 20. Bivariate binary logistic regression analysis was performed to determine each of explanatory variables and variables with p value less than 0.2 during bivariate analysis were entered to multivariate analysis. Multivariate binary logistic regression analysis was conducted to determine the presence of a statistically significant association between explanatory variables and outcome variables. P values less than 0.05 were considered statistically significant and strength of the association was presented using adjusted odds ratio with 95% CI.

## 3. Results

### 3.1. Sociodemographic Characteristics of the Participants

A total of 415 participants were included in the study with the response rate of 98.1%. The mean age of the respondents was 34.56 (±12.65) with age ranging from 15 to 78 years. Majority, 222 (53.5%), were males. Of the total participants, 161 (38.8%) were orthodox religion follower and 134 (32.3%) were Amhara in their ethnicity. The educational status of participants indicated that 118 (28.4%) of them attended primary level of education. Large numbers of respondents were from urban 328 (79%). The monthly income of respondents ranged from 100 to 25000 Ethiopian birr and majority, 246 (59.3%), have monthly income less than 1539ETB (Table 1).

### 3.2. Clinical, Psychosocial, and Substance Related Factors of the Respondents

Regarding the clinical characteristics of the respondents, the majorities were with the diagnosis of pulmonary tuberculosis 344 (83%). More than half of the participants were in new treatment category 257 (61.9%) and two-thirds of the participants were in intensive phase of treatment 277 (65.5%). Among participants, 30 (7.2%) reported family history of suicide. Comorbid depression was reported by 129(31.1%) of participants. Poor social support and perceived TB stigma were reported by 177 (42.7%) and 187 (45.1%) of participants, respectively. From all study participants, 40 (9.6%) had cocomorbid HIV illness. Current substance use was reported by 60 (14.5%) of participants (Table 2).

### 3.3. Life Time Prevalence of Suicidal Ideation and Attempt among Patients with Tuberculosis

Suicidal ideation was reported by 72 (17.3%) with 95%CI, 13.7-20.6, of the respondents in their life time. The life time prevalence of suicidal attempt among participants was 31(7.5%) with 95% CI, 4.8-10.4. Regarding frequency of the attempt, 20 (64.5%), 8 (25.8%), and 3 (9.7%) of participants had attempted suicide once, twice, and more than two times, respectively. The most commonly used method of an attempt was hanging 14 (45.2%) followed by use of overdoes of antituberculosis medications, 9 (29%). More than half suicidal attempters, 17 (54.8%), reported that their suicidal attempt was related to current physical illness. Among respondents who attempted suicide 20 (64.5%) of participants seriously attempted to kill themselves (Table 3).

### 3.4. Factors Associated with Suicidal Ideation and Suicidal Attempt

To identify the effect of each variable, multicollinearity statistics test was done and the variance inflation factor (VIF) was in the range of 1.05 to 2.5 for all variables. Results of the final logistic regression analysis indicated that being female AOR=2.7; 95%CI (1.39-5.23) and having comorbid depression AOR=4.9, 95%CI (2.56-9.40) were significantly associated with both suicidal ideations and attempt. Respondents with no formal education were about 3 times AOR= 3.35, 95%CI (1.26-8.91) more likely to have suicidal ideation. Perceived tuberculosis stigma and being diagnosed as extra-pulmonary TB were also significantly associated with suicidal ideation AOR=3.24, 95%CI (1.64-6.44) and AOR=2.35, 95%CI (1.1-4.98), respectively (Table 4).

But treatment category of multidrug resistant tuberculosis AOR=3.06, 95%CI (1.23-7.6) and comorbid HIV illness AOR=6.7, 95%CI (2.24-19.94) were specifically significantly associated with suicidal attempt (Table 5).

## 4. Discussions

The current study showed that the magnitude of suicidal ideation was 17.3% with 95% CI (13.7-20.6). Regarding the magnitude, the finding of the current study was similar to studies carried out in Benin and Korea which was reported to be 16% and 21%, respectively [11, 26].

The finding of current study was lower than study done in Nigeria 44.3% [13] and United States 25.2% [10]. The possible reason for the discrepancy might be variation in study design and sample size used. The Nigerian study used case control study design and the US study was a survey among medically ill patients. There were also differences in study participant and study setting where the study participants were from general chronic physical illness treatment centers in US and the Nigerian study included both inpatients and outpatient participants. Another possible reason might also socioeconomic and cultural differences between the previous and the currents study.

However, it was higher than studies conducted in South Africa 9% [12] and India 9% [15]. The variation might be due to difference in study design in which community based survey was conducted in a study from South Africa and difference in study participants that only include patient on direct observation short course therapy (DOTS) in India. Participants on DOTS had daily contact with professional and their interaction could minimize suicidal related behaviors.

Among factors significantly associated with suicidal ideation, being female was about 2.7 times more likely to have suicidal ideation than males. This is supported by study results from South Africa [12] and Benin [27]. This might be due to cultural influence in which women might not discuss their problems as men and the suicidal ideation may be due to their suppressed emotion. Another possible reason might be related to depression in which females are two times more prone to depression as compared to males [28]. Participants who have no formal education were 3.35 times more likely to have suicidal ideation. This might be due to lack of knowledge about treatment outcome of tuberculosis and considering it as life ending disease and fear of being discriminated by the society [29]. Diagnosis of extra pulmonary tuberculosis was another factor associated with suicidal ideation. This is in agreement with study done from Nigeria [13]. It could be due to the reason that extra pulmonary tuberculosis is more pain full and has poor prognosis than pulmonary tuberculosis. As result, it might lead patients to think about suicide more [30]. Patients who have comorbid depression were about 5 times more likely to have suicidal ideation. This is supported by the study conducted in South Africa [12], Nigeria [13], and India [15]. The possible reason might be that depression decreases neurotransmitter serotonin which resulted in suicidal behavior. Regarding perceived TB stigma, patients with perceived TB stigma were 3.24 times more likely to have suicidal ideation. This is in agreement with study conducted in India [15]. Patients with perceived TB stigma might have low self-esteem and social isolation and these factors might predispose them to develop suicidal ideation [31].

The magnitude of suicidal attempt in this study was 7.5% with 95% CI (4.8-10.4) which is consistent with survey study conducted in United States which reported 8.9% of attempted suicide [10]. It was higher than studies conducted in South Africa 3.1% [12], Korea 2.6% [11], and Italy 0.5% [27]. It was lower than the case-control study result conducted in Nigeria which was 14.8% [13]. The variation might be due to difference in study design, sample size, use of different assessment tools, and difference in study participants.

Being female was 4.57 times more likely to have suicidal attempts. Similar result was obtained in a study conducted in Benin [12]. It is also consistent with the global WHO suicide report that indicated females attempt more suicide than males but committed less than males [4]. The possible reason for this might be women's greater vulnerability to other psychosocial stressors. Participants with treatment category of multidrug resistant tuberculosis (MDR-TB) were 3.06 times more likely to attempt suicide than new non-MDR TB respondents. This finding is supported by previous study conducted from Nigeria [13]. It is known that patients with drug resistant TB have persistent illness, pill burden, and longer ongoing stressors. These factors and the severity and nature of TB category itself might contribute a lot about patients' hopelessness of recovery. Regarding depression, participants with depression were 4.75 times more likely to have suicidal attempt. This is consistent with other studies in South Africa and Nigeria [12, 13]. The possible reason may be in patients with depression the magnitude of suicide due to the overwhelming distress of the illness itself being generally high. In this kind of people the neurotransmitter serotonin is generally low and studies have shown that decreased level of serotonin result in suicidal behavior [32]. Another factor which was strongly associated with suicidal attempt was presence of comorbid HIV illness. Participants with comorbid HIV illness were 6.67 times more likely to have suicidal attempts as compared to HIV negative patients. It was consistent with other studies [12, 13]. The possible reasons for this might be due to nature of the disease (HIV), effect of associated medical illnesses, being diagnosed with HIV which is terminal life-long disease associated with high level of stigma, and increased vulnerability to high rate of mental disorders [33].

While there are important findings in the current study, care should be taken in interpreting the results taking in to consideration the following limitation. Recall bias and social desirability bias due to the nature of measurement tools used and use of interviewer administered questionnaire to obtain information could be considered as limitation of this study. Since the entire sample was taken from one hospital found in the capital city, findings of this study might not be generalized to other areas especially in rural settings.

## 5. Conclusions

In the current study the magnitude of suicidal ideation and attempt were high as compared to general population and majorities of other studies. Both suicidal ideation and attempt had statistically significant with female sex and comorbid depression. Participants who have no formal education, diagnosis of extra pulmonary tuberculosis, and perceived TB stigma were significantly associated with suicidal ideation. Comorbid HIV illness and having multidrug resistant tuberculosis (MDR-TB) treatment category were significantly associated with suicidal attempt. Therefore, the current study area and other settings which provide TB screening and treatment needed to assess patients for suicidal ideation and attempt and provide intervention giving more emphasis on patients with risk factors. Additionally, further research using different study design on risk factors of suicidal ideation and attempt should be conducted to broaden the current findings.

## Figures and Tables

**Table 1:** Sociodemographic characteristics of TB patients attending outpatient treatment at Saint Peter**'**s Specialized Hospital, Addis Ababa, Ethiopia, 2018.

Variable	Frequency(N=415)	Percent (%)
Age		
15-24	96	23.1
25-34	140	33.74
35-44	83	20
45-54	61	14.7
>54	35	8.4

Sex		
Male	222	53.5
Female	193	46.5

Religion		
Orthodox	162	39
Muslim	116	28
Protestant	95	22.9
Catholic	29	7
Others*∗*	13	3.1

Marital status		
Married	176	42.4
Single	140	33.7
Divorced	54	13
Widowed	45	10.8

Ethnicity		
Amhara	134	32.3
Oromo	117	28.2
Tigre	91	21.9
Gurage	56	13.5
Others*∗∗*	17	4.1

Education status		
Have no formal education	79	19
Primary	118	28.4
Secondary	94	22.7
Preparatory	63	15.2
College and above	61	14.7

Occupational status		
Farming	97	23.4
Trading	135	32.5
Government employed	101	24.34
Private employed	62	14.94
Others*∗∗∗*	20	4.82

Residency		
Urban	328	79
Rural	87	21

Average monthly income		
<1539 ETB	246	59.3
≥1539ETB	169	40.7

Others *∗*=Jehovah's witnesses and no religion, *∗∗*= Wolyita and silte *∗∗∗*=house wife and students.

**Table 2:** Clinical, psychosocial, and substance related factors of TB patients attending outpatient treatment at Saint Peter's Specialized Hospital, Addis Ababa, Ethiopia, 2018.

Variables	Frequency	Percent (%)
Site affected by TB		
Pulmonary	344	83
Extra-pulmonary	71	17

Duration of illness		
<6 months	166	40
6-12 months	185	44.6
>12 months	64	15.4

Category of TB treatment		
New	257	61.9
MDR-TB	105	25.3
Re-treatment	53	12.8

Phase of treatment		
Intensive phase	272	65.5
Continuation phase	143	34.5

Co-morbid physical illness		
HIV	40	9.6
Other chronic illness	33	8
No	342	82.4

Family Hx of mental illness		
Yes	53	12.8
No	362	87.2

Family Hx of suicide		
Yes	30	7.2
No	385	92.8

Co-morbid depression		
Yes	129	31.1
No	286	68.9

Social support		
Poor	177	42.7
Moderate	121	29.4
Strong	117	28

Perceived TB stigma		
Yes	187	45.1
No	228	54.9

Substance use (alcohol, khat &cigarette)		
Yes	60	14.5
No	355	85.5

**Table 3:** Suicidal ideation and attempt among TB patients attending outpatient treatment at Saint Peter**'**s Specialized Hospital, Addis Ababa, Ethiopia, 2018.

variables	Frequency	Percent (%)
Ever suicidal ideation		
Yes	72	17.35
No	343	82.65

Suicidal ideation in 1 month		
Yes	41	9.88
No	374	90.12

Ever plan of suicide		
Yes	54	13
No	361	87

Ever suicide attempt		
Yes	31	7.5
No	384	92.5

Suicidal attempt in 1 month		
Yes	13	3.1
No	402	96.9

Frequency of suicide attempt		
Once	20	64.5
Twice	8	25.8
More than two times	3	9.7

Reason for suicidal attempt		
Physical illness(TB)	17	54.8
Family conflict	9	29
Death of family	4	13
Puberty	1	3.2

Severity related to attempt		
Seriously attempted	20	64.5
Methods used not effective	10	32.3
To seek help	1	3.2

Methods of attempt		
Hanging	14	45.2
Drug overdoes	9	29
Poisoning	5	16.1
Sharp tools	3	9.7

**Table 4:** Factors associated with suicidal ideation of participants attending outpatient treatment at Saint Peter's Specialized Hospital, Addis Ababa, Ethiopia, 2018.

Explanatory variables	Suicidal ideation		COR, (95%CI)	AOR, (95%CI)
	Yes(N)	No(N)		
Sex				
Male	25	197	1	1
Female	47	146	2.54,(1.49,4.31)	*2.7(1.39, 5.23)∗∗∗*

Educational status				
Have no formal education	33	46	3.26, (1.48, 7.2)	*3.35,(1.26, 8.91)∗*
Primary	10	108	0.42, (0.17,1.06)	0.41,(0.142, 1.2)
Secondary	11	83	0.60, (0.24,1.5)	0.51,(0.173, 1.47)
Preparatory	7	56	0.57, (0.21,1.58)	0.57,(0.172, 1.85)
College and above	11	50	1	1

Site of TB				
Pulmonary	45	299	1	1
Extra-pulmonary	27	44	4.07(2.3, 7.23)	*2.35,(1.1, 4.98)∗*

Category of TB Rx				
New	41	219	1	1
MDR	27	80	1.8(1.04, 3.12)	1.38, (0.69, 2.8)
Re-treatment	4	44	0.5(0.165, 1.43)	0.37,(0.10, 1.41)

Family mental illness				
Yes	14	39	1.88(0.96,3.69)	2.2,(0.85, 5.8)
No	58	304	1	1

medical illness				
HIV	12	28	2.3(1.10, 4.77)	1.12,(0.38, 3.2)
Others	6	27	1.18(0.46, 3)	0.61, (0.18, 2.05)
No	54	288	1	1

Depression				
Yes	47	82	5.98(3.47,10.32)	*4.9,(2.56, 9.40)∗∗∗*
No	25	261	1	1

Social support				
Poor	45	132	2.73(1.4, 5.32)	1.2,(.52, 2.73)
Moderate	14	107	1.05(0.47, 2.34)	0.64, (0.24, 1.7)
Strong	13	104	1	1

Perceived TB stigma				
Yes	53	134	4.35(2.47, 7.67)	*3.24,(1.64, 6.44)∗∗∗*
No	19	209	1	1

*Others* =diabetics, hypertension, and cardiac disease; Chi square=5.5, df=8, and Hosmer-Lemeshow test p value= 0.661.

*∗* = p value<0.05; *∗∗∗* = p value <0.01.

**Table 5:** Factors associated with suicidal attempt of participants attending outpatient treatment at Saint Peter's Specialized Hospital, Addis Ababa, Ethiopia, 2018.

Explanatory variables	Suicidal Attempt		COR, (95%CI)	AOR, (95%CI)
	Yes(N)	No		
Sex				
Male	7	215	1	1
Female	24	169	4.36,(1.84,10.37)	*4.57 (1.7, 12.27)∗∗∗*

Site of TB				
Pulmonary	19	325	1	1
Extra-pulmonary	12	59	3.48(1.6, 7.55)	1.6,(0.6, 4.17)

Category of TB Rx				
New	13	247	1	1
MDR	16	91	3.34(1.55, 7.22)	*3.06, (1.23, 7.6)∗*
Re-treatment	2	46	0.83(0.18, 3.78)	0.324,(0.05, 2.28)

Family HX mental illness				
Yes	8	45	2.62(1.11,6.21)	2.4,(0.7, 8.27)
No	23	339	1	1

Co-morbid medical illness				
HIV	12	28	8.2 (3.56, 18.86)	*6.7,(2.24, 19.94)∗∗∗*
Others	2	31	1.2(0.27, 5.58)	0.64, (0.12, 3.56)
No	17	325	1	1

Co-morbid depression				
Yes	21	108	5.4(2.45, 11.77)	*4.75,(1.89, 11.91)∗∗∗*
No	10	276	1	1

Social support				
Poor	23	154	3.35(1.23, 9.07)	2.81,(0.89, 8.93)
Moderate	3	118	0.55(0.13, 2.44)	0.66, (0.13, 3.3)
Strong	5	112	1	1

Perceived TB stigma				
Yes	19	168	2.04(0.961,4.311)	1.12,(0.45, 2.76)
No	12	216	1	1

*Others* =diabetics, hypertension, and cardiac disease; Chi square=4, df=8, and Hosmer-Lemeshow test p value = 0.84.

*∗* = p value<0.05; *∗∗∗* = p value <0.01.

## Data Availability

The data included in the manuscript can be accessed from corresponding author upon request by the email alexmolla09@gmail.com.

## Supplementary Materials

Supplementary Materials Figure 1: frequencies of social support among patients with tuberculosis visiting outpatient clinics at Saint Peter's Hospital, Addis Ababa, Ethiopia, 2018(N=415). Figure 2: percentage of perceived tuberculosis stigma among patients with tuberculosis visiting outpatient clinics at Saint Peter's Hospital, Addis Ababa, Ethiopia, 2018(N=415). Figure 3: numbers of suicidal attempt among patients with tuberculosis visiting outpatient clinics at Saint Peter's specialized hospital, Addis Ababa, Ethiopia, 2018. Figure 4: distributions of current substance use among patients with tuberculosis at Saint Peter's Hospital, Addis Ababa, Ethiopia, 2018 (N=415).
