# Supplementary material for: Suicidal Ideation, Attempt, and Associated Factors among Patients with Tuberculosis in Ethiopia: A Cross-Sectional Study
Source: Psychiatry J. 2019 Mar 13;2019:4149806. doi: 10.1155/2019/4149806 (PMC6436369; doi:10.1155/2019/4149806)
Supplement: Supplementary Materials — Figure 1: frequencies of social support among patients with tuberculosis visiting outpatient clinics at Saint Peter's Hospital, Addis Ababa, Ethiopia, 2018(N=415). Figure 2: percentage of perceived tuberculosis stigma among patients with tuberculosis visiting outpatient clinics at Saint Peter's Hospital, Addis Ababa, Ethiopia, 2018(N=415). Figure 3: numbers of suicidal attempt among patients with tuberculosis visiting outpatient clinics at Saint Peter's specialized hospital, Addis Ababa, Ethiopia, 2018. Figure 4: distributions of current substance use among patients with tuberculosis at Saint Peter's Hospital, Addis Ababa, Ethiopia, 2018 (N=415). [file 4149806.f1.docx]

# Supplementary materials

# Frequencies of social support among patients with tuberculosis

# Figure 1: Frequencies of social support among patients with tuberculosis visiting outpatient clinics at Saint Peter`s Hospital, Addis Ababa, Ethiopia, 2018(N=415)

# Percentage of perceived stigma among patients with tuberculosis

# Figure 2: Percentage of perceived tuberculosis stigma among patients with tuberculosis visiting outpatient clinics at Saint Peter`s Hospital, Addis Ababa, Ethiopia, 2018(N=415)

1. Frequencies of suicidal attempt among patients with tuberculosis

Figure 3: Numbers of suicidal attempt among patients with tuberculosis visiting outpatient clinics at Saint Peter`s specialized hospital, Addis Ababa, Ethiopia, 2018.

1. Frequencies of current substance use(Alcohol, khat and cigarette) among patients with Tuberculosis

**Figure: 4** Distributions of current substance use among patients with tuberculosis at Saint peter`s hospital, Addis Ababa, Ethiopia, 2018(N=415)
